# Bronchoscopic resection of typical carcinoid tumors: literature review and case series

**DOI:** 10.1093/jscr/rjac180

**Published:** 2022-05-17

**Authors:** Salman Alahmed, Hassan Arishi, Mohammed Alabdulatif, Hana Bamefleh, Raed Alghamdi

**Affiliations:** Divion of General Surgery, Department of Surgery, King Abdulaziz Medical City, National Guard Health Affairs, Riyadh, Saudi Arabia; Divion of Thoracic Surgery, Department of Surgery, King Abdulaziz Medical City, National Guard Health Affairs, Riyadh, Saudi Arabia; Divion of Thoracic Surgery, Department of Surgery, King Abdulaziz Medical City, National Guard Health Affairs, Riyadh, Saudi Arabia; Department of Pathology and Laboratory Medicine, King Abdulaziz Medical City, National Guard Health Affairs, Riyadh, Saudi Arabia; Divion of General Surgery, Department of Surgery, King Abdulaziz Medical City, National Guard Health Affairs, Riyadh, Saudi Arabia

## Abstract

Carcinoid tumors have been treated with formal oncological surgical resection, which is considered the gold standard approach. In the past two decades, bronchoscopic has gained popularity for treatment of carcinoid tumors. A 34-year-old female with an unresolving chronic cough, underwent a chest CT that showed right endobronchial lesion. Bronchoscopy and a biopsy were taken from the lesion; pathology confirmed the diagnosis to be a typical carcinoid tumor. Forty-six-year-old male, who was a smoker, suffered from hemoptysis. Pathology revealed it to be an endotracheal typical carcinoid tumor at the carina. Uncertainties, such as chances of recurrence, cost-effectiveness and the inability to achieve formal oncological resection have been discussed and challenged in the literature. With gaining use of this treatment approach, tissue-sparing surgery for carcinoids can improve patient quality of life both in the short and long term.

## INTRODUCTION

Tracheal and bronchial typical and atypical carcinoid tumors, belonging to the subset of thoracic neuroendocrine tumors that also is comprised of small cell lung carcinoma and large cell neuroendocrine carcinoma, have had a paradigm shift in its treatment approach; moving towards tissue-sparing endobronchial resections with close monitoring from the gold standard of formal surgical oncological resections [1]. Referring to the World Health Organization’s latest lung tumor classification, typical carcinoids differ from atypical by showing less mitotic activity, less than two mitoses per 2 mm^2^ versus 2–10 mitoses per 2 mm^2^, as well as having a lower potential for necrosis, lymphatic invasion and metastasis. Both tend to have an endobronchial location, either centrally or peripherally [2]. Many surgical approaches have been described for the resection of typical and atypical carcinoid tumors, ranging from surgical bronchoplasty for bronchial carcinoids to pneumonectomies for atypical carcinoids [3]. Brokx *et al*. presented an algorithm for management of carcinoid that starts initially with bronchoscopic treatment, advocating a tissue sparing approach, and arguing against extensive surgical resection early on, pointing towards the lack of evidence to support the notion of advancement of the disease to the point where a more radical resection is warranted, given the slow-growing nature carcinoids in general. The authors reported a 42% rate of complete remission in a sample of 112 patients with carcinoid cancer; with a disease-specific 5-year survival rate of 100% following their approach [4]. Endobronchial resection using electrocautery, cryotherapy and laser photoresection, all have been utilized to reach the goal of complete resection. In this paper, we present two cases of typical carcinoid, occurring in a male and female patient, both presented with hemoptysis and both underwent endobronchial resection using electrocautery.

## CASE SERIES

**Figure 1:**
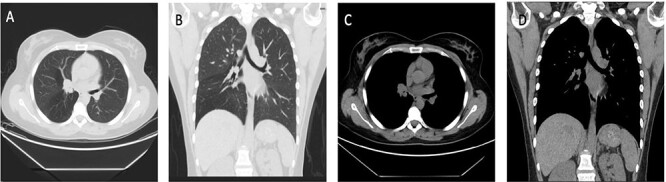
Case 1: (**A** and **B**) Lung window showing endo-bronchial lesion at the right bronchus intermedius with air trapping middle and lower lobes. (**C** and **D**) Same lesion evident on mediastinal window on both axial and coronal cuts.

A 34-year-old female, who is a known case of ulcerative colitis, has been followed by a pulmonologist due to a chronic cough, that was first treated as asthma; but did not resolve with the management plan set by the pulmonology service. A CT scan (Fig. 1) was then conducted on this patient that showed right endobronchial lesion. Afterwards, this patient underwent bronchoscopy and a biopsy was taken from the cherry-like lesion mentioned. Pathology confirmed the diagnosis to be a typical carcinoid tumor (Fig. 1; case 1). The decision was made by the treating team to take the patient for tissue-sparing endobronchial resection. During the operation, the lesion was identified in the right intermedius bronchus, and by the use of a bronchoscopic snare and electrocautery, it was separated and removed en bloc.

**Figure 2:**
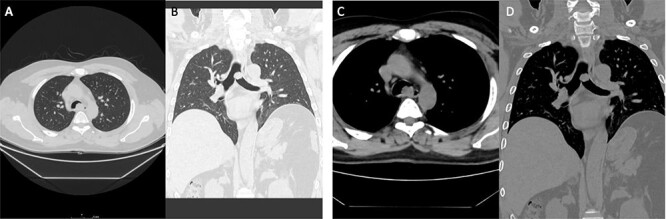
Case 2: panels (**A** and **B**) show an intratracheal soft tissue mass just above the carina more toward the left side. (**C** and **D**) Same lesion on axial cuts with radiological evidence of infiltration/invading left lateral wall of the trachea.

The second case involved a 46-year-old male, who was a smoker for 15 years, however, had quit smoking for 1 year prior to presentation, otherwise medically and surgically free. He had a main complaint of periodic hemoptysis. This patient’s CT scan (Fig. 2) showed a 2.2 × 2 cm lesion at the level of the carina. Operatively, the lesion was resected en bloc in a similar fashion to the first portrayed case, using a combination of bronchoscopic snare and electrocautery. Pathology revealed an endotracheal typical carcinoid tumor (Fig. 1; case 2).

Both patients were followed 3 months later with a repeat bronchoscopy, which was clear from any signs of recurrence.

## PATHOLOGY

See Fig. 3.

**Figure 3:**
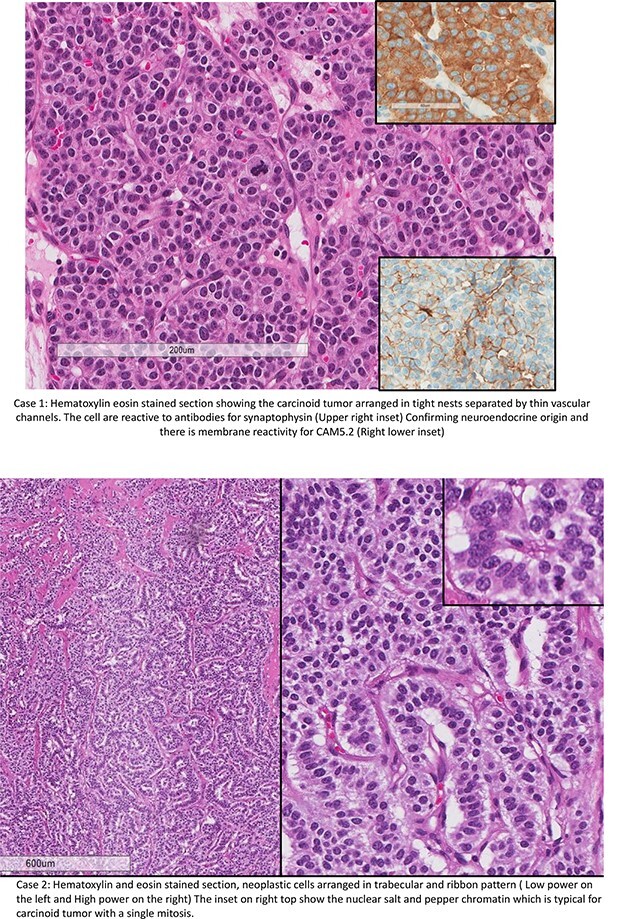
Case 1: hematoxylin eosin stained section showing the carcinoid tumor arranged in tight nests separated by thin vascular channels. The cells are reactive to antibodies for synaptophysin (upper right inset). Confirming neuroendocrine origin and there is membrane reactivity for CAM5.2 (right lower inset). Case 2: hematoxylin and eosin stained section, neoplastic cells arranged in trabecular and ribbon pattern (low power on the left and high power on the right). The inset on right top shows the nuclear salt and pepper chromatin, which is typical for carcinoid tumor with a single mitosis.

## DISCUSSION

The use of a tissue-sparing resection strategy for endotracheal and endobronchial carcinoid tumors has been challenged by the notion of inability to achieve an oncological; that is by surgical lymph node resections and by resecting beyond the tumor’s gross borders, satisfying the possibility of an extraluminal tumor growth. For the past two decades, questions have been raised regarding initial bronchoscopic resection of carcinoid tumors, such as whether a less aggressive approach to treatment would lead to a worse outcome later on if recurrence occurs, and whether this strategy would be less cost-effective without a definite clear advantage to the gold standard oncological surgical resection. Surveillance and follow up algorithms also play a crucial role in patients undergoing initial bronchoscopic resection, and a consensus has yet to be set.

Van Boxem *et al*. reported a success rate of 74% in a group of nineteen patient who underwent bronchoscopic resection, with the remainder undergoing radical surgical resection upon follow up. The authors suggest following patients utilizing a high-resolution CT scan to assess for extraluminal extension after initial bronchoscopic resection in patients who are fit for minimally invasive debulking surgery [5].

Brokx *et al*. published findings of a prospective long-term study after initial bronchoscopic resections of carcinoid tumor that showed a recurrence rate of 7.8% in a group of 51 patients who were diagnosed 47–198 months after initial resection, noting the distant metastasis in some. However, the author argued that the decision of initial bronchoscopic treatment did not compromise the later need for radical surgical resection [4].

Typical carcinoids have a low tendency to metastasize to lymph nodes, unlike atypical carcinoids; 3–20% versus 48–75%, respectively [6]. Since the nature of carcinoid tumors are typically considered to be slow-growing, the trend towards curative minimally invasive initial resection is warranted, reasoning that there is abundant time for follow up with high-resolution CT scans and bronchoscopies, and possible resolution to a more aggressive surgical resection if needed without compromising patients’ outcome.

Dewan *et al*. concurs on the initial tissue-sparing strategy; however, raising the point that this might not be suitable in developing countries, where patients usually present late with a more complicated disease progression in comparison with the experiences of surgeons in more developed countries. Feasibility of follow-up can also be questioned in the absence of a technologically advanced health care system [7].

In Saudi Arabia, surgical strategies vary when dealing with carcinoid tumors. While metropolitan cities can follow a tissue-sparing strategy with ease considering the availability of specialized thoracic surgeons, technologically advanced tertiary care hospital networks, and a health-aware patient population, the same cannot be generalized to more rural areas where access to niche surgical care may be limited. Taking this into consideration, it is integral to inquire about patients’ access to healthcare and general background before opting for initial bronchoscopic resection with close follow-up. Moreover, follow-up algorithms have yet to be well described in the aforementioned strategy, which also presents itself as a challenge in this developing movement of tissue-sparing surgery. Mode of resection being by endoscopic electrocautery, snare resection, the use of a laser modality have not been compared in terms of recurrence, whether any would have an effect on recurrence and achieving adequate excision. It is exciting to consider tissue-sparing resection being the mainstay of treatment considering the benefits it offers the patient when it comes to limiting post-operative complications that accompany radical surgical resections, improving quality of life and hastening return to daily activities. Prospective studies need to be performed in a multicentric fashion to be able to fully answer the questions around this bold approach to treatment of carcinoid tumors.

## CONCLUSION

Carcinoid tumors that are endotracheal and endobronchial managed with bronchoscopic resection showed promising post-operative and short to medium term results in decreasing patient morbidity and allowing for quicker recovery; with gaining popularity of this approach, there is hope in a robust long term follow-up model to streamline tissue-sparing surgery for carcinoids of the trachea and bronchus.

## AUTHORS’ CONTRIBUTIONS

S.A. had the main role of literature review and analysis of relevant papers, writing the manuscript and supervision of all related roles in producing this study. H.A. initiated the study, revised and reviewed the manuscript. M.A. had an advisory role and is the director of this initiative of tissue-sparing surgery in Saudi Arabia. R.A. aided in writing the manuscript, gathering data and final review.

## CONFLICT OF INTEREST STATEMENT

The authors declare that there are no competing interests in regards to this paper.
